# Finite element analysis of dental implants with validation: to what extent can we expect the model to predict biological phenomena? A literature review and proposal for classification of a validation process

**DOI:** 10.1186/s40729-018-0119-5

**Published:** 2018-03-08

**Authors:** Yuanhan Chang, Abhijit Anil Tambe, Yoshinobu Maeda, Masahiro Wada, Tomoya Gonda

**Affiliations:** 1 Department of Prosthodontics, Gerodontology and Oral Rehabilitation, Osaka University Graduate School of Dentistry, 1-8 Yamadaoka, Suita, Osaka, 565-0871 Japan; 2 Mahatma Gandhi Vidyamandir’s Karmaveer Bhausaheb Hiray Dental College & Hospital, Mumbai Agra Road, Panchwati, Nashik, Maharashtra India

**Keywords:** Finite element analysis, Dental implant, Validation, Verification

## Abstract

A literature review of finite element analysis (FEA) studies of dental implants with their model validation process was performed to establish the criteria for evaluating validation methods with respect to their similarity to biological behavior. An electronic literature search of PubMed was conducted up to January 2017 using the Medical Subject Headings “dental implants” and “finite element analysis.” After accessing the full texts, the context of each article was searched using the words “valid” and “validation” and articles in which these words appeared were read to determine whether they met the inclusion criteria for the review. Of 601 articles published from 1997 to 2016, 48 that met the eligibility criteria were selected. The articles were categorized according to their validation method as follows: in vivo experiments in humans (*n* = 1) and other animals (*n* = 3), model experiments (*n* = 32), others’ clinical data and past literature (*n* = 9), and other software (*n* = 2). Validation techniques with a high level of sufficiency and efficiency are still rare in FEA studies of dental implants. High-level validation, especially using in vivo experiments tied to an accurate finite element method, needs to become an established part of FEA studies. The recognition of a validation process should be considered when judging the practicality of an FEA study.

## Review

### Background

Finite element analysis (FEA) has been applied to investigate dental implant designs, the structure and material of the superstructure, and the stability of the surrounding bone [1, 2]. According to PubMed, only 10 FEA studies of dental implants were published in 1990, while 102 papers were published in 2014.

FEA has become an increasingly useful tool in the past few decades. In the medical field, the behavior of any structure or tissue under a particular stimulation can be evaluated using FEA, and biomechanical changes in the tissues can be analyzed. Additionally, FEA allows for measurement of the stress distribution inside of the bone and various dental implant designs during mastication; such measurements are impossible to perform in vivo [1, 2, 3]. 

A large number of FEA regarding dental implant and bone were published in these decades; however, the precision and accuracy of those studies in silico are still questionable. In 2009, Dumont et al. [4] indicated that FEA studies of biological structures should be validated experimentally whenever possible. Hannam [5] stated that the minimum requirements of FEA studies should include comparisons with data from other work or any data that can be gleaned from the living subjects being modeled.

According to the American Society of Mechanical Engineers Committee on verification and validation in computational solid mechanics, verification is defined as “the process of determining that a computational model accurately represents the underlying mathematical model and its solution,” while validation is defined as “the process of determining the degree to which a model is an accurate representation of the real world from the perspective of the intended uses of the model.” In simple terms, verification is the process of “solving the equations right,” whereas validation is the process of “solving the right equations” [6–9]. Validation is a process by which computational predictions are compared with experimental data in an effort to assess the modeling error [6–9]. The sole purpose of these “experiments” is to produce data for comparison with model predictions rather than to address specific scientific hypotheses.

FEA studies with validation have recently become more common in the biomechanical field. FEA validations can be divided into two types: (1) direct validation, which involves experiments on the quantities of interest (from basic material characterizations to hierarchical system analysis such as model experiments and in vitro experiments), and (2) indirect validation, which involves the use of literature or the results of previous clinical studies. Indirect validation is clearly less favored than direct validation because of its uncertain experimental quality, sources of error, and high degree of variability. However, indirect validation may be unavoidable in FEA because no concrete biological outcome can be directly attributed to most FEA studies of force distribution; thus, it is difficult to generate outcome data for comparison with experimental data. Therefore, FEA studies should include a validation method to prove the close similarity of the results to the actual clinical situation. Validation is the process of “solving the right equations” [6–9] and comparing computational predictions with experimental data (the “gold standard”) in an effort to assess the modeling error.

The purpose of this literature review of FEA studies was to examine their model validation process and establish the criteria for evaluating validation methods with respect to their similarity to biological behavior.

### Materials and methods

All studies included in this review (eligibility criteria) were FEA studies of the stress distribution of dental implants and surrounding bone using any type of validation method, and all were published in English. The exclusion criteria were publication in a language other than English, appearance of the word “validation” only in the references, no mention of the validation method for numerical FEA analysis, and mentioning of the requirement for validation without conduction of the actual validation.

**Fig. 1:**
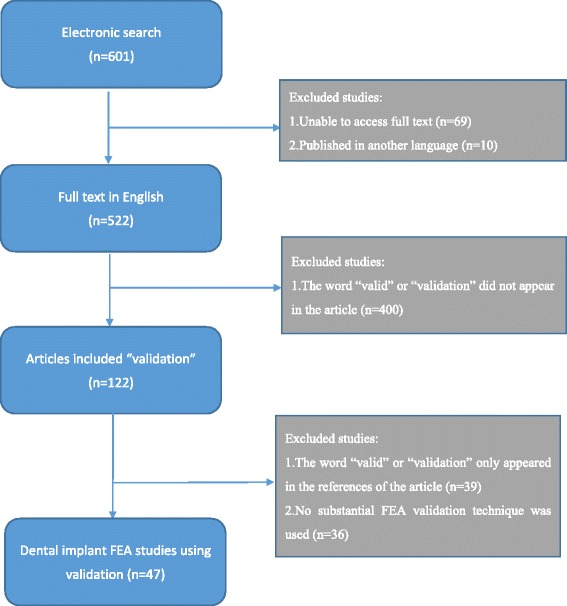
Flowchart of literature review. An electronic literature search of PubMed was conducted up to January 2017 using the Medical Subject Headings “dental implants” and “finite element analysis.” After accessing the full texts, the context of each article was searched using the words “valid” and “validation” and articles in which these words appeared were read to determine whether they met the inclusion criteria for the review

An electronic literature search of PubMed was conducted up to January 2017 using the Medical Subject Headings “dental implants” and “finite element analysis.” After accessing the full text, the full context was searched using the words “valid” and “validation,” and all articles containing these words were read to determine whether they met the inclusion criteria. The selected articles were then read and summarized, and the validation techniques used in each article were assessed and categorized in a hierarchy (Fig. 1).

### Results

**Fig. 2:**
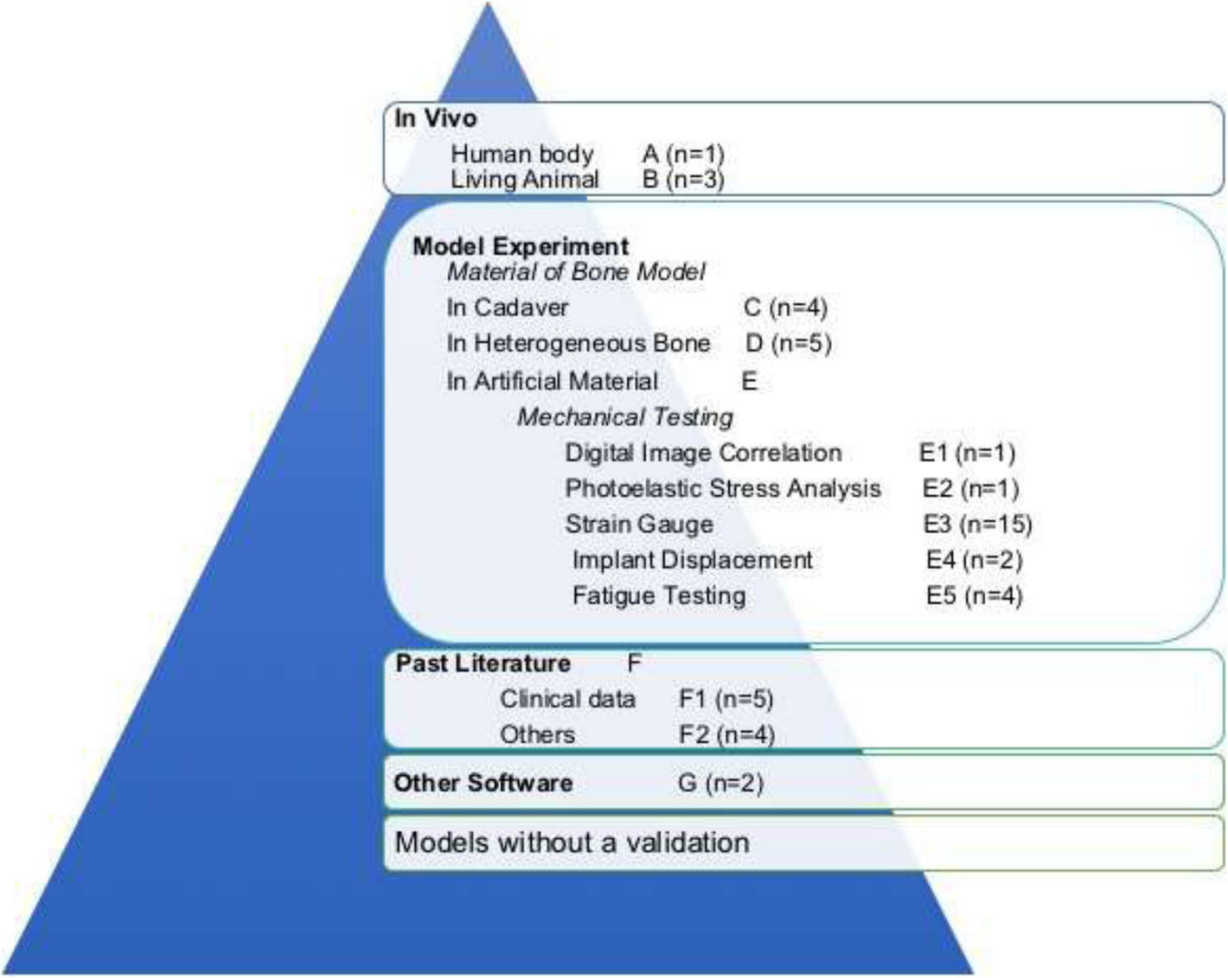
Hierarchy of validations based on their similarity to real biomechanical behaviors. The articles (*n* = 47) were categorized according to their validation method as follows: in vivo experiments in humans (*n* = 1) and other animals (*n* = 3), model experiments (*n* = 32), others’ clinical data and past literature (*n* = 9), and other software (*n* = 2)

**Fig. 3:**
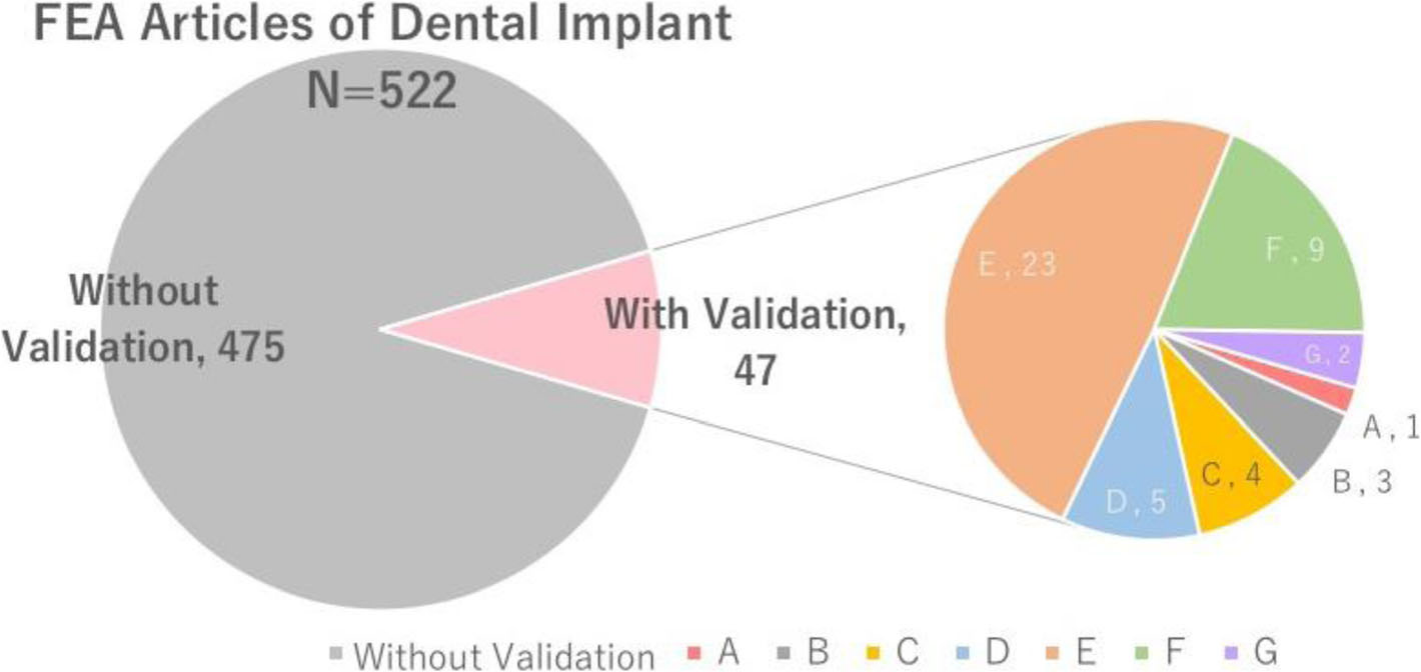
Proportion of dental implant FEA articles with a validation. (*Left*) Among totally 522 FEA articles of dental implants which we were able to access English full text up to January 2017, there are only 47 articles with a validation. (*Right*) The articles with a validation were categorized according to their validation method as follows levels: A, in vivo (human bodies); B, performed in vivo (heterogeneous animals); C, model experiment performed using part of a cadaver; D, model experiment performed using heterogeneous bone; E, model experiment performed using artificial materials; F, comparison with past literature; and G, performed with other software (*n* = 2)

In total, 601 articles were obtained from the PubMed electronic search using the Medical Subject Headings “dental implants” and “finite element analysis.” After excluding articles for which the full text could not be accessed (*n* = 69) and that were not written in English (*n* = 10), 522 articles remained. These articles were searched using the terms “validation,” “validity,” and “valid” to determine whether the studies had performed a validation; after this process, 122 articles remained. These 122 articles were read, and 47 that met the eligibility criteria were selected and are summarized in Table 1. These articles were all FEA studies published from 1997 to 2016. The articles were categorized according to the method of validation as follows: in vivo experiments, model experiments, use of others’ clinical data or literature, and other software (Figs. 2 and 3).

**Table 1:** All studies in the literature that considered with an actual validation of FEA

Ranking	Authors	Year	FE model	FEM geometry reference	Material properties of tissues around implants	Validation	Material of validation model	Comparison items
A	Heckmann et al. [10]	2006	Implants embedded in a bone block	CAD: bone block with a cortical layer and cancellous layer was constructed by CAD	Homogeneous isotropic linear elasticity: cortical and trabecular bone	(1) In vitro testing: strain gauge on implant support bridge in resin bone model (2) In vivo testing: strain gauge on pontic of a 3-unit bridge in humans	(1) Epoxy resin (2) In vivo: in a patient’s mouth	Surface strain of resin/resin
B	Hou et al. [12]	2009	Implants embedded in bone (rat’s mouth)	CT: CT data of the implant in a rat model	Not mentioned	In vivo experiment: implants placed in rat, and histologic findings compared after loading	In vivo: rat	Histologic findings
B	Natali et al. [11]	1997	Implants embedded in a bone block	CAD: bone section constructed by CAD	Homogeneous isotropic linear elasticity: cortical and trabecular bone	In vivo experiment: implant insertion in animal (dog) for loading and creation of sections of bone and implants	In vivo: dog	Visualization of change in bone and stress analysis by FEA
B	Cha et al. [13]	2015	Implant engaged in bone disc (model was used to calculate torque)	CAD: based on histology of the bone–implant interface	Homogeneous isotropic linear elasticity	In vivo experiment: implant insertion in animal (mice) with different insertion torques	In vivo: mice	Histomorphometric analyses
C	Nagasao et al. [16]	2009	Implants embedded in normal mandibles and reconstructed mandible (with fibulae or ribs) and under mastication movement (dynamic condition)	CT: dry mandibles, ribs, and fibulae	Homogeneous isotropic linear elasticity: cortical and cancellous bone of every part of mandible, fibula, and rib	Mechanical testing: implant embedded in 3 full mandibles and surface strain under loading measured by strain gauge	Dry mandible as mandible	Surface strain of bone under same conditions in FEA/experiment
C	Nagasao et al. [17]	2010	Implants embedded in normal mandibles and reconstructed mandible (with fibulae or ribs) under mastication movement (dynamic condition)	CT: dry mandibles, ribs, and fibulae	Homogeneous isotropic linear elasticity: cortical and cancellous bone of every part of mandible, fibula, and rib	Mechanical testing: implant embedded in 2 full mandibles and surface strain under loading measured by strain gauge	Dry mandible as mandible	Surface strain of bone under same conditions in FEA/experiment
C	Eser et al. [18]	2009	Four implants embedded in the maxilla with bar superstructure	CAD: model of nonanatomic maxilla, individualized arch form according to implant alignment	Homogeneous isotropic linear elasticity: cortical bone, cancellous bone, Ti, Alloy, bar-superstructure	Ex vivo strain gauge measurement of cadaver’s maxilla (with implants)	Cadaver	Surface strain of bone (maxilla)
C	Nagasao et al. [19]	2006	Implants embedded in normal maxilla and cleft maxilla	CT and CAD: normal maxilla: CT from a dry skull; palatal cleft, alveolar cleft, and complete cleft were designed by computer	Homogeneous isotropic linear elasticity: cortical and trabecular bone	Mechanical testing: strain measurement by strain gauge and implant embedded in actual skull model	Dry skull	Surface strain of bone
D	Bardyn et al. [20]	2010	Implants embedded in bone (polyurethane foam and sheep bone)	CT: polyurethane foam block and sheep bone	Nonhomogeneous: calculated from CT data	Mechanical testing in both polyurethane foam and sheep bone: measurement of removal torque of the implant	Polyurethane foam and sheep bone	Removal torque of implants
D	Olsen et al. [21]	2005	Implants embedded in porcine mandibles from CT data and application of loading on the implant of FEM	CT: porcine mandibles	Nonhomogeneous: calculated from CT data	Mechanical testing: comparison of displacement with actual measurements under the same testing load	Block of porcine mandible	Implant displacement under loading
D	Huang et al. [22]	2002	Implant embedded in bone block	CAD: bone block model constructed by CAD	Homogeneous isotropic linear elasticity: cortical and trabecular bone	In vitro model testing experiments: implant in bone cubic and measurement of resonance frequencies	Bone section from lumbar vertebrae of hogs	Value of resonance frequency
D	Hasan et al. [23]	2012	Implant (implant and abutment together) embedded in bovine bone	CT: scan of the models used for the experiment (implant embedded in bovine rib)	Homogeneous isotropic linear elasticity: bovine cortical bone, bovine cancellous bone	Mechanical tests: implant displacement and rotation under loading were measured using a biomechanical measurement system (laser pinhole and camera)	Bovine rib section as mandible bone	Displacement of the abutment
D	Chatzigianni et al. [24]	2011	Mini-implant embedded in bone	CT: scan of the specimen used for the experiment (implant embedded in bovine rib)	Homogeneous isotropic linear elasticity: bovine cortical bone, bovine cancellous bone	Mechanical tests: implant displacement and rotation under loading were measured using a 3D mobility measurement system (laser beams and camera)	Bovine rib section as mandible bone	Displacement of the abutment
E1	Tiossi et al. [14]	2013	Implants and tooth (acrylic) embedded in resin block model, crowns (splint and non-splint)	CAD: epoxy model block	Nil (in this FEM, there was a resin block only and no living tissue simulation)	Digital image correlation (DIC): images of deforming body captured and strain calculated. Mechanical testing with implants embedded in resin block	Resin block as mandible bone	Calculated surface strain by DIC and FEA
E2	Ozçelik et al. [25]	2007	Three-unit bridge fixed prosthesis (with rigid connector and non-rigid) supported by an implant and a natural tooth, with an adjacent tooth and surrounding bone	CAD: a bone section (2D) was constructed by CAD with a cortical layer and spongious bone and PDL	Homogeneous isotropic linear elasticity: enamel, dentin, pulp, cortical bone, cancellous bone	Photoelastic stress analysis methods (PSAM): implants placed in photoelastic resin, then force loaded and photograph taken	Photoelastic resin as bone	Stress distribution in bone/resin
E3	Chou et al. [26]	2014	A section of mandible and implant	CT data and 2D FE model used in previous study	Homogeneous isotropic linear elasticity	Mechanical testing: implant embedded in resin bone and strain measured by strain gauge	3D printer to build acrylic-based polymer	Surface strain of bone
E3	Mobilio et al. [29]	2013	Implant embedded in a bone block	CAD: bone block built by CAD with a cortical (1.5 mm) and trabecular (28.5 mm) layer	Homogenous anisotropic linear elasticity cortical bone: orthotopic linearly elastic material; trabecular bone: transversely isotropic linearly elastic material	Mechanical testing: implant embedded in resin block and strain measured by strain gauge	Resin block as mandible bone	Load and strain relationship
E3	Chang et al. [30]	2012	Short implants with crowns embedded in left posterior segment of maxilla	CT: CT scan of a dry human male skull	Homogeneous isotropic linear elasticity: cortical bone, cancellous bone (high and low density)	Mechanical testing: strain measured by strain gauge and implant embedded in resin block under loading	ABS plastic bone as maxillary bone	Surface strain of bone/resin
E3	Tu et al. [31]	2010	Implant embedded in resin block	CAD: a resin block with a cortical layer and cancellous layer was constructed by CAD	Nil (in this FEM, there was a resin block only and no living tissue simulation)	Mechanical testing: strain measured by strain gauge and implant embedded in resin mandible section	Resin bone as mandible bone	Surface strain of bone/resin
E3	Lin et al. [32]	2010	Implant embedded in the left maxilla with crown	CT of intact healthy male patient	Homogeneous isotropic linear elasticity: cortical bone, cancellous bone	Mechanical testing: strain measured by strain gauge and implant embedded in resin mandible section	ABS plastic bone as maxillary bone	Surface strain of bone/resin
E3	Qian et al. [33]	2009	Implant embedded in bone block	CAD: a bone cubic with cortical layer and cancellous layer was constructed by CAD	Homogeneous isotropic linear elasticity: cortical bone, cancellous bone	(1) In vitro experiment: mechanical testing with resin bone and digital image correlation to calculate displacement of implant and strain on bone (2) Literature data: strain gauge measurement in model experiment	Resin block as mandible bone	(1) Displacement of implant and strain on bone (2) Surface strain of bone
E3	Karl et al. [34]	2009	Implant embedded in base made by 3 materials	CAD: acrylic, G10 epoxy resin, aluminum	Homogeneous isotropic linear elasticity: acrylic, G10 epoxy resin, aluminum	Mechanical testing: strain gauge. FEA-calculated strain was compared with strain gauge results	Acrylic resin, glass-filled epoxy, aluminum	Surface strain of acrylic resin, glass-filled epoxy, aluminum
E3	Hsu et al. [35]	2009	Implant embedded in resin block (with resin’s parameter for consistence with experiment)	Nil	Homogeneous isotropic linear elasticity: Resin (epoxy and Tempron)	Mechanical testing: implant embedded in resin bone section and surface strain under loading was measured by strain gauge	Resin block as mandible bone	Surface strain of resin/resin
E3	Nagasawa et al. [36]	2008	Implant embedded in a bone block (only compact bone)	CAD: a bone block (compact bone) was constructed by CAD	Homogeneous isotropic linear elasticity: compact bone	Mechanical loading test for implant, sectioned longitudinally	Nil	Implant deformation; no scientific values
E3	Huang et al. [37]	2005	Splinted or non-splinted 2-unit crowns supported by 2 or 3 implants embedded in bone	CT: CT of posterior portion of a cadaver mandible	Homogeneous anisotropic linear elasticity cortical bone: orthotopic linearly elastic material; trabecular bone: transversely isotropic linearly elastic material	Mechanical test: strain measured by strain gauge on model	Acrylic resin as mandible bone	Surface strain of resin/bone
E3	Iplikçioğlu et al. [38]	2003	Implant embedded in bone block	CAD: a resin block model was constructed by CAD	Nil (in this FEM, there was a resin model only and no living tissue simulation)	Mechanical test: measurement of stress on the implant, abutment, and resin	Resin block as bone	Stress distribution in resin and implants
E3	Chang et al. [27]	2016	Ball attachment overdenture (mandible, implant and attachment, mucosa, denture)	CT: from a single human mandible (edentulous 65-year-old woman)	Homogeneous isotropic linear elasticity	Mechanical test: strain measured by strain gauge on surface of bone model	Rapid prototype ABS plastic bone model, and a 3-mm layer of silicone to simulate mucosa	Surface strain of resin/bone
E3	Rezende et al. [28]	2015	Bone section with embedded implant and prosthesis (metal coping and porcelain), screws	CT: in vitro model (resin bone)	Homogeneous isotropic linear elasticity	Mechanical test: strain measured by strain gauge on surface of bone model	Polyurethane resin	Surface strain of resin/bone
E3	Chang et al. [39]	2012	Implants embedded in maxilla section with imperfect and perfect osseointegration under force loading	CT: data of maxillary first molar area	Inhomogeneous anisotropic linear elasticity cortical bone: anisotropic Trabecular bone: transversely isotropic linearly elastic material	Mechanical testing: strain measured by strain gauge and implant embedded in resin block	Resin block as maxillary bone	Surface strain of bone/resin block
E3	Chang et al. [40]	2012	Implants and crowns in a section of the maxilla	CAD: a bone block with a cortical layer and cancellous layer was constructed by CAD	Homogeneous anisotropic linear elasticity compact bone, cancellous bone	Mechanical testing: strain measured by strain gauge and implant embedded in resin block	ABS resin block as mandible bone	Surface strain of bone/resin block
E4	Zhiyong et al. [41]	2004	(1) Single tooth in bone block (2) Single implant in bone block (3) Various FPD supported by tooth and implant	CAD: a bone block model was constructed by CAD	Homogeneous isotropic linear elasticity: cortical and trabecular bone, dentin, PDL	Mechanical testing: comparison of displacement with actual measurements under the same tested loading conditions	Not mentioned	Implant displacement under loading
E4	Chang et al. [42]	2012	Implant embedded in a bone block	CAD: a bone block with a cortical layer and cancellous layer was constructed by CAD	Homogeneous isotropic linear elasticity: compact bone, cancellous bone	Mechanical testing: pullout testing of mini-implant inserted in synthetic bone material	Synthetic bone material as mandible bone	Pullout strength of mini-implant
E5	Inglam et al. [43]	2013	Implant embedded in a bone block	CAD: a bone block with a cortical layer and cancellous layer was constructed by CAD	Homogeneous anisotropic linear elasticity cortical bone: orthotopic isotropic Trabecular bone: transversely isotropic linearly elastic material	Mechanical testing: strain measured by strain gauge and implant embedded in resin block	Resin block as mandible bone	Surface strain of bone/resin block
E5	Necchi et al. [44]	2003	Implant (fixture, abutment, and connecting screw)	Nil	Nil	Mechanical failure tests: preloading and functional loading conditions	Not mentioned	Maximum breaking force
E5	Genna et al. [45]	2003	Implant embedded in bone block	CAD: a resin block model was constructed by CAD	Nil (in this FEM, there was a resin model only and no living tissue simulation)	Cyclic mechanical fatigue testing: implant placed in epoxy resin and section of specimen examined under microscope	Epoxy resin block as bone	Comparison of locations of stress focus
E5	Perriard et al. [46]	2002	Different types of implant bodies and abutments embedded in resin	CAD: epoxy resin as bone	Nil (in this FEM, there was a resin model only and no living tissue simulation)	Mechanical fatigue testing of implant model: until half of samples still survived under loading	Resin block	Comparison of locations of stress concentrations
F1	Bruno Salles Sotto-Maior et al. [47]	2016	A bone model of mandibular right posterior region	CT: from a patient’s mandible	Homogeneous isotropic linear elasticity: cortical and trabecular bone	Clinical findings of bone loss at 1-year follow-up	In vivo: radiographic films of patients	Mechanoregulatory tissue model was employed to monitor the morphological changes in bone subjected to biomechanical loading
F1	Wang et al. [48]	2013	A 3D model of maxillary bone	CT image of maxillary bone section missing both central incisors	Homogeneous isotropic linear elasticity	Radiographs qualitatively compared regarding resemblance between computational remodeling results and clinical data	In vivo: radiographic films of patients	Comparison of variations in maxillary bone densities
F1	Choi et al. [50]	2012	Implants embedded in anterior maxilla	CT: anterior maxillary bone	Homogeneous isotropic linear elasticity compact bone, cancellous bone	Comparison of model implant displacement under the same load with clinical outcomes in literature	Literature	Model implant displacement
F1	Shen et al. [51]	2010	Implant embedded in mandibular right first molar area	CAD: a bone block with a cortical layer and cancellous layer was constructed by CAD	Homogeneous anisotropic linear elasticity: cortical bone, cancellous bone	Clinical data; comparison of implant displacement value under 20-N loading from clinical data	Clinical results	Implant displacement under loading
F1	Lin et al. [52]	2010	Implant embedded in mandible (cortical and cancellous bone), crown, teeth	CT: in vivo CT of a segment of mandible	Inhomogeneous anisotropic linear elasticity: cortical bone, cancellous bone (properties varied with density)	Clinical data: comparison of bone density with other clinical follow-up X-ray images	X-ray images of human	X-ray images
F2	MacGinnis et al. [49]	2014	3D skull model with masked sutures	CT: from 42-year-old man, 3D skull image excluding the mandible	Homogeneous isotropic linear elasticity	Comparison with past literature		Comparison with conclusions of past literature
F2	Fanuscu et al. [53]	2004	Unilateral edentulous posterior maxilla with grafted sinus	CAD: unilateral edentulous posterior maxilla with grafted sinus was constructed by computer	Homogeneous isotropic linear elasticity: cortical and trabecular bone	Validation with previous study by one of the authors in which photoelastic modeling with similar geometry was used		Location of stress
F2	Mellal et al. [54]	2004	Cylindrical implant, bone consisting of a cancellous core coated with cortical envelope	CAD: a bone section model was constructed by CAD	Homogeneous isotropic linear elasticity: cortical and trabecular bone	Literature: systematic search of the literature was conducted to relate the numerical predictions to existing in vivo data		
F2	Zarone et al. [55]	2003	Mandible with 6 implants and prosthetic superstructure	Laser: a man’s total mandible by laser digitizer	Homogeneous isotropic linear elasticity: cortical and trabecular bone	Data from previous experiments: comparisons of range of medial convergence during opening and protrusive movements		
G	Bulaqi et al. [56]	2015	Implants embedded in a bone block	CT data: mandible	Homogeneous isotropic linear elasticity	Comparison with theoretically predicted values (calculated with the equations)		values of conical to wretch torque ratio
G	Vayron et al. [57]	2015	Implants embedded in a bone block	CAD: cortical bone, newly formed bone, and trabecular bone	Homogeneous isotropic mechanical properties	Comparison with results using a 2D finite difference numerical model

Based on the validation methods described in the articles, the top portion of the validation hierarchy comprised studies that used a customized clinical method in a human for validation [10]. The next level of the hierarchy comprised studies that used models for validation, including animal models [11–13] and mechanical experiments. Mechanical experiments were divided according to the material used for bone models and the techniques used for testing those models. The materials were divided into homogenous bone, heterogeneous bone, and artificial materials such as acrylic, polyurethane, plastic bone material, and others. Various validation methods were used in studies that employed mechanical testing of bone models using these specific artificial materials, such as digital image correction [11], photo-elastic stress analysis [15], and use of a strain gauge test attached to a model (this was the most commonly used method, described in 15 of 48 articles). These techniques also involved measurement of the implant displacement and fatigue testing of an implant body. The next level of the hierarchy comprised studies that used literature or clinical data from other articles to compare with results of FEA. The final level comprised studies that used other computer software for support but did not perform an actual experiment.

We classified all validation processes based on their similarity to real biomechanical behaviors into the following hierarchy (levels A to G) (Fig. 2):

- Level A: performed in vivo (human bodies) (*n* = 1) [10]

The top level of the hierarchy, level A, includes in vivo methods of FEA validation conducted in humans. In 2006, Heckmann et al. [10] quantified the degree of stress that occurs in the bone around the implants as a result of fixation of cemented and screw-retained fixed partial dentures. They used a computer-aided design (CAD) model of an implant embedded in a bone block for FEA, and strain gauge experiments were performed under the same loading conditions with the use of a resin bone model and a human being for validation.

- Level B: performed in vivo (heterogeneous animals) (*n* = 3) [11–13]

Three studies conducted animal experiments for FEA validation. In 2009, Hou et al. [12] conducted an FEA validation study involving rats to assess the histological change in the mechanical environment surrounding loaded and unloaded implants. In 1997, Natali et al. [11] performed a validation study in which they compared the influence of axial and nonaxial forces on the bone tissue surrounding oral implants placed in dogs. Both research groups used computed tomography data and CAD techniques to create an FEA model. Similarly, in 2015, Cha et al. [13] used murine femurs to place implants with low and high insertion torques for FEA validation.

- Level C: model experiment performed using part of a cadaver (*n* = 4) [16–19]
- Level D: model experiment performed using heterogeneous bone (*n* = 5) [20–24]

The next two levels in the hierarchy comprised in vivo model experiments on a section of a cadaver (level C) and the bone of heterogeneous animals (level D). Most of these studies involved mechanical testing, such as recording strain by a strain gauge attached to a dry skull or a section of bovine, porcine, or sheep bone. Bardyn et al. [20] compared the FEA-predicted removal torque with that measured using sheep bone and polyurethane foam as a validation technique. Olsen et al. [21] scanned a porcine mandible to create an FEA model and compared the FEA-predicted implant displacement with that measured on the same porcine mandible as a validation technique. Additionally, in 2002, Huang et al. [22] determined the vibrating behavior of a dental implant under various surrounding bone conditions using bone sections from hogs and FEA. The resonance frequency was compared between the two techniques, but in this case, FEA seemed more likely to serve as a validation technique to support the results of the model experiment.

- Level E: model experiment performed using artificial materials (*n* = 23) [14, 25, 26–46]

Artificial materials such as acrylic resin, polyurethane, or plastic bone models were commonly used as embedded “bone” implants in validation experiments. Level E includes the use of special materials and specific methods to measure the force distribution and photoelastic resin as well as a technique called digital image correlation described by Tiossi et al. [14] in 2013. Comparisons of these artificial materials is difficult because it is challenging to determine how much more accurate one technique is over another technique. Even after subcategorizing the techniques from E1 to E5, we found that no one technique was superior to any other.

- Level F: performed by comparison with past literature (*n* = 9) [47–55]

Validations in this level involve comparison of FEA with clinical data (F1) or other literature (F2). Most such studies compared FEA with “similar” conditions in patients, but either the comparisons were not customized or indirect and ill-defined clinical results (e.g., bone resorption volume in length or radiographic X-ray images) were compared with force in FEA. Level F2 includes validation using past literature with similar results or conclusions that were mostly summarized in few words in the “Discussion” section of an article.

- Level G: performed by comparison with other software (*n* = 2) [56, 57]

The last level, level G, includes validation performed by another type of computer software such as two-dimensional FEA, i.e., an FEA model built in a computer is validated by another computer simulation or calculated values.

### Discussion

The use of FEA for dental implants and surrounding bone has increased during the past few decades. Our PubMed search using the terms “dental implants” and “finite element analysis” revealed about 450 papers published in the past 10 years. However, FEA studies of implants using validation experiments are comparatively rare. While prior studies had effectively outlined the importance of validation in biomechanical FEA, no reviews of studies that applied validation to computational biomechanics of dental implants have been performed.

Table 1 shows all studies in the literature that considered the need for validation of FEAs. According to these studies, we established a hierarchy based on the evidence level of the validations (A to G, i.e., high to low) (Fig. 2).

Level A: validation using living humans

Level B: validation using living heterogeneous animals

Levels C and D: validation using homogenous and heterogeneous bone

Level E: validation using artificial bone materials

Level F: validation using past literature

Level G: validation using other software

FEA using model verification cannot be considered to be a validation method for entire study. Model verification should be performed to ensure accurate FEA; however, finite element models verified with clinical data such as a patient’s computed tomography findings are categorized in a low level of validation or without validation. For this reason, studies that used only model verification (some studies may called it by "model validation") were not included in our review [58–62].

Many recent papers [10–12, 14, 15, 25–31, 33, 35, 36, 39, 41–45, 47, 48, 50–52, 54, 55, 58–74] have described the use of FEA to evaluate the stress distribution of implant fixtures and surrounding bone, with a particular focus on different fixture lengths, shapes, connection designs, and prostheses. However, most such studies [15, 58–74] were performed without validation executed under the same conditions with the FEA. The following questions are worthy of consideration by oral scientists and clinicians: Can a finite element model really create a virtual condition simulating the biomechanical behavior of the craniomandibular system? To what extent can we predict biological activities with finite element models [9]?

The complexity of living organisms and internal biological phenomena is impossible to fully and precisely duplicate with individual-level specificity using a computer. However, we can evaluate the limitations of current technology and build a model with the highest level of evidence possible.

Because of the limitations of computer technology, most FEA models [75–79] simplify the skeletal muscle architecture in terms of a uniform fiber length, pennation angle, and line of action and represent the architecture using a Hill-based muscle model. However, how well the modeling of skeletal muscles as one-dimensional strings represents the behavior of the full three-dimensional muscles remains unknown. Reducing the complexity of the muscles to strings entirely neglects the variations in muscle density (deformation) and structure during the complex movement of real muscle specimens, which is difficult to acquire.

This review focused on validation of FEA and established a hierarchy of validation techniques from high to low as a reference for further FEA studies. However, due to the limitations of this study, the boundary conditions and finite element method (FEM) settings were not considered. For example, some research may have involved high-level validation performed in vivo, but the original FEM model was built by CAD using only a simple flat two-layer bone and without any model verification. Some other studies used a simulated bone (computed tomography data from homogeneous, heterogeneous, or artificial materials) as an FEM geometry reference and performed the validation on that material only, without seeking to perform validation using a more realistic material. Both the use of a detailed, accurate model that closely resembles the real condition and the performance of validation to prove its accuracy are important. As computer technology has progressed, model verification has become more sophisticated and complicated; however, validation still should not be ignored.

While conducting this review, we also considered future efforts. There are two types of FEA studies: time-dependent studies, which have a validity period within which the process must take place, and time-independent studies, which have no validity period but only analyze the stress distribution at a single point in time. To date, several biomechanical studies have been published with time-independent analysis [10–12, 14, 15, 25–31, 33, 35, 36, 39, 41–45, 47, 48, 50–52, 54, 55, 58–74, 80] (e.g., examination of bone resorption underneath the denture base, analysis of the instant stress distribution of a dental implant, and the bones or components of an artificial knee joint). Maeda and Wood [80] simulated a bone-dependent bone resorption process using an FEM model and shape-optimization algorithm.

To explain or analyze the mechanical properties involved in biological phenomena such as motor tasks (mastication, walking, or heart contraction), a time-dependent finite element model may provide a more realistic view. However, if time-dependent performance criteria are considered (the most common is to clarify the influence of musculoskeletal structure on function or the performance of a motor task), dynamic optimization and boundary conditions are required. This means that a much more complex model including many parameters and properties must be built, despite some of these real-world physiological data being unknown. This difficulty may explain why time-dependent models of mastication for FEA are rare.

## Conclusions

1. High-level validation of FEA using in vivo experiments is still rare in the dental implant field.
2. It is necessary to clearly indicate the validation process of the model when a study using FEA is presented.
3. The hierarchy proposed in this study based on the evidence level of the validations can be applied to evaluate the clinical significance of studies using FEA.
